# HERV-E-Mediated Modulation of PLA2G4A Transcription in Urothelial Carcinoma

**DOI:** 10.1371/journal.pone.0049341

**Published:** 2012-11-07

**Authors:** Darko Gosenca, Ute Gabriel, Annette Steidler, Jens Mayer, Olivia Diem, Philipp Erben, Alice Fabarius, Christine Leib-Mösch, Wolf-Karsten Hofmann, Wolfgang Seifarth

**Affiliations:** 1 Department of Hematology and Oncology, Mannheim Medical Center, University of Heidelberg, Mannheim, Germany; 2 Department of Urology, Mannheim Medical Center, University of Heidelberg, Mannheim, Germany; 3 Department of Human Genetics, Center of Human and Molecular Biology, Medical Faculty, University of Saarland, Homburg, Germany; 4 Institute of Virology, Helmholtz Zentrum München, German Research Center for Environmental Health, Neuherberg, Germany; University of Oxford, United Kingdom

## Abstract

Human endogenous retroviruses (HERV) and related elements account for more than 8% of the human genome and significantly contribute to the human transcriptome by long terminal repeat (LTR) promoter activity. In this context, HERVs are thought to intervene in the expression of adjacent genes by providing regulatory sequences (cis-effect) or via noncoding RNA including natural antisense transcripts. To address the potential impact of HERV activity in urothelial carcinoma, we comparatively analyzed the HERV transcription profiles in paired samples of non-malignant urothelium and urothelial carcinoma derived from 13 patients with bladder cancer by means of a retrovirus-specific microarray (RetroArray). We established a characteristic HERV signature consisting of six ubiquitously active HERV subgroups (E4-1, HERV-Rb, ERV9, HERV-K-T47D, NMWV3, HERV-KC4). The transcription pattern is largely identical in human urothelial carcinoma, non-malignant urothelial tissue, four tumor-derived cell lines and in a non-malignant urothelial cell line (UROtsa). Quantitative reverse transcriptase PCR (qRT-PCR) of HERV-E4-1, HERV-K(HML-6) and HERV-T(S71-TK1) revealed a bias to lower HERV activity in carcinoma samples compared to non-malignant tissue. Determination of active HERV-E4-1 loci by cloning and sequencing revealed six HERV-E4-1 proviral loci that are differentially regulated in urothelial carcinoma cells and normal tissue. Two full-length HERV-E4-1 proviruses, HERV-Ec1 and HERV-Ec6, are located in antisense orientation in introns of the genes PLA2G4A and RNGTT, respectively. PLA2G4A encodes a cytosolic phospholipase A2 (cPLA2) that is dysregulated in many human tumors. PLA2G4A and HERV-Ec1 displayed reciprocal transcript levels in 7 of 11 urothelial carcinoma patients. Moreover, reciprocal shifts were observed after treatment of UROtsa cells with HERV-Ec1 and PLA2G4A-directed siRNAs or 5-aza-2′-deoxycytidine (aza-dC) pointing to an antagonistic regulation of PLA2G4A and HERV-Ec1 transcription in human urothelial cells. We suggest that transcription of HERV-Ec1 contributes to fine tuning of cPLA2 expression, thereby facilitating tumorigenesis.

## Introduction

Constituting at least 40% of the human genome it is becoming increasingly evident that genetic mobile elements are an integral part of the transcriptional regulatory machinery of the cell and can influence gene expression at transcription and protein levels [1], [2], [3], [4], [5], [6]. Since molecular pathogenesis of all human cancers concurs with alterations in gene and/or gene product activities [7], an involvement of transposable elements (TEs) in carcinogenesis was repeatedly postulated [8], [9], [10]. TE activities in human cancers may reflect pathogenic alterations in disease development or may even actively contribute to pathogenesis by dysregulation of gene expression [11], [12]. Human TEs have been considered to contribute tens of thousands of natural antisense transcripts to human genes [13]. Therefore, their role in regulatory mechanisms such as RNA interference (RNAi) and epigenetics needs to be investigated in both healthy and malignant cells.

LTR retrotransposons including human endogenous retroviruses (HERVs) constitute about one fifth of human TEs and 8–9% of the human genome. Contributing a high number of promoters to gene regulatory networks they have a long history not only as potential pathogens but also as a source of genetic variation, genome evolution, and gene regulation [1], [3], [13], reviewed in [4], [6], [14], [15], [16]. Whereas some LTR elements such as mammalian apparent LTR retrotransposons (MaLR) represent ancient retrotransposons, class I, II and III HERVs are considered to be remnants of germ line infections by exogenous retroviruses that were endogenized and genetically fixed in the human population [17], [18], [19] (reviewed in [14]). In the course of evolution endogenous retroviruses have been amplified several times and thus spread throughout the genome by repeated events of retrotransposition and/or reinfection [20].

Although HERVs are noninfectious, replication-defective retroviral relics, at least some proviruses of each HERV group were found to be still transcriptionally active [21]. In addition, many solitary HERV LTRs, remnants of internal recombination events, have preserved their promoter activity and still contain active regulatory elements such as enhancer sequences, transcription factor binding sites or polyadenylation signals [4], [22], [23]. A bioinformatical analysis revealed tens of thousands of active retroviral promoters in the human genome [2]. According to this approach transcribed HERV sequences were derived from 1.16% of the human genome and all transcripts that initiate from LTRs were found to cover 22.4% of the human genome sequence. Moreover, HERV-LTR-initiated antisense transcripts may modulate the corresponding sense transcript levels and thus influence gene expression [24]. In some cases, HERVs have adopted physiological “symbiotic” functions (reviewed in [6], [25], [26]).The probably most prominent example of HERV importance for physiological functions is the expression of Env proteins (Syncytin1 and 2) in the placenta that play an essential role in cell-fusion during syncytiotrophoblast formation [27] and maternofetal tolerance [28]. HERV activity has been detected in all human tissues and organs investigated so far [21], [29]. Furthermore, altered HERV activity is detected in many tumors when compared to corresponding normal tissues (reviewed in [30], [31]. Germ-cell tumors, in particular seminomas show a high frequency of HERV activity and HERV-K(HML-2) encoded Gag proteins were identified in seminoma biopsies [32], [33]. In some cancers, HERVs have been shown to encode highly immunogenic epitopes that can be considered as a new class of tumor-specific antigens [34], [35], [36]. A HERV-K(HML-2) Gag-related antigen was detected in prostate cancer and an antibody response against the recombinant protein was also observed in bladder cancer patients [37].

Bladder cancer is a widely spread and life threatening disease (worldwide: 9th most common tumor [38]). In western countries, urothelial carcinoma is the most common type (>90%) of bladder cancer. To date, no comprehensive study of HERV expression in non-malignant urothelium (N) and urothelial carcinoma (T) has been performed. However, hypomethylation of TEs in human urothelial carcinomas suggests a possible activation of retroelements including HERVs [39].

To address the potential association of HERV activity with urothelial cancer, we comparatively analyzed the transcriptional activity of HERVs in samples of non-malignant urothelium and urothelial carcinoma by means of a powerful microarray based assay (RetroArray) [21], [40], [41]. This microarray comprises 50 representative HERV RT-derived sequences from 20 major HERV groups that correspond to HERV families described formerly in literature. We used the term “family” in previous publications but replaced it now by “group” since “family” is already reserved as taxonomic definition for *Retroviridae* [42]. We report on HERV transcription signatures found in bladder tissue specimen representing pairs of non-malignant and malignant samples from 13 patients. HERV-E4-1, HERV-K(HML-6) and HERV-T(S71-TK1) subgroups were further analyzed in a total of 52 primary samples by qRT-PCR. Active HERV-E4-1 loci were determined by cloning and sequencing of qRT-PCR amplicons and their potential interaction within the genomic context is discussed. Using UROtsa cells as an *in vitro* model of the normal urothelium, HERV-E4-1-dependent modulation of cytosolic phospholipase A2 (PLA2G4A) transcript levels was investigated by means of HERV-E LTR-directed siRNAs and aza-dC treatment.

## Materials and Methods

### Ethics Statement

Non-malignant urothelium and urothelial carcinoma tissue samples were collected from patients treated at the Department of Urology of the University Hospital Mannheim. A positive vote was obtained from the Ethic Committee II, Faculty of Medicine, University of Heidelberg (Vote ID: 2007-030N-MA) for tissue collection and data acquisition for further investigations from patients being treated at the Department. Written informed consent was obtained from all patients included in the study.

### Human Urothelial Tissue Samples

Tissues were collected from routine tumor surgery and immediately placed in RNAlater^©^. Tissues were stored in RNAlater^©^ at 4°C over night and were then snap frozen in liquid nitrogen and stored at −80°C until further processing. Tumor staging was based on the current classification of urothelial neoplasms [43].

### Cell Lines and aza-dC Treatment

The four human cancer cell lines, UMUC-3 (American Type Culture Collection, ATCC, USA), RT-112 (German Collection of Microorganisms and Cell Cultures, DSMZ, Braunschweig, Germany), HT-1197 and T24/83 (both European Collection of Cell Cultures, ECACC, Salisbury, UK) and the normal urothelial cell line UROtsa [44], [45] were cultivated as described previously [46]. In general, cell culture media were supplemented with 10% fetal calf serum and penicillin-streptomycin at a concentration of 100 µg/ml. All cells were routinely tested for mycoplasma contamination using LookOut Mycoplasma PCR Detection Kit (Sigma-Aldrich, Steinheim, Germany). Cells were treated with 5 µM aza-dC (Sigma-Aldrich) for 48 h. The concentration was non-cytotoxic as determined by a colorimetric MTT assay in 96 well plates as described [47]. As controls, untreated cells of the same passage were cultivated in parallel for each experiment.

### RNA Preparation and First Strand cDNA Synthesis

**Figure 1:**
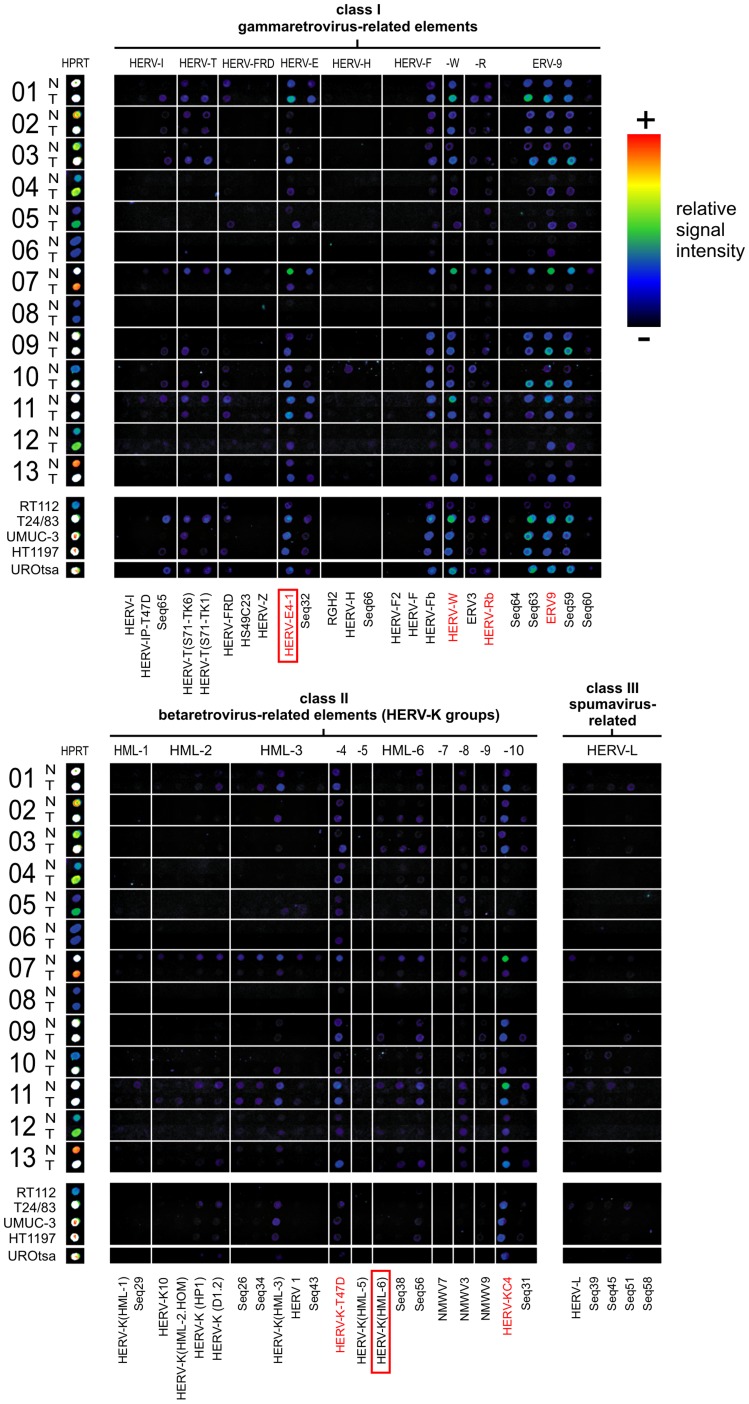
HERV transcription activity in patient samples by RetroArray analysis. HERV activity profiles representing pairs of malignant (T) and non-malignant (N) urothelial tissue specimen, each pair derived from the same patient (n = 13) were digitally aligned. Below, the HERV signatures of four urothelial cancer cell lines (RT112, T24/83, UMUC-3, HT1197) and of the non-malignant urothelial cell line UROtsa are shown. HERV subgroups representing the urothelial core profile are emphasized with red letters. The housekeeping gene hypoxanthine phosphoribosyl transferase (HPRT) served as internal control. For detailed information about the identity of targets and capture probes, see Table S1 and references [21], [41]. Each positive spot on the microarray represents multiple HERV loci assigned to one subgroup of multicopy elements with sufficient sequence similarity so that individual elements cannot be distinguished. Although false color mapping was used for improved image visualization, weak signals may be unrecognizable in the figure. QRT-PCR was performed for HERV-E4-1, HERV-T(S71-TK1) and HERV-K(HML-6), as depicted by red boxes.

Frozen tissue samples and cells harvested from 6-well dishes were processed using RNeasy MiniKit (QIAGEN, Hilden, Germany). Tissue samples (3 to 30 mg) were mechanically disrupted in RLT buffer by TissueLyser (QIAGEN). Except for patients with advanced invasive tumors undergoing cystectomy (n = 10) normal tissue samples were limited to less than 1 µg RNA due to operational standard practices and ethical reasons. The reduced RNA yields of normal tissue limited the number of possible experiments and influenced signal strength of RetroArray as demonstrated by weaker signals for housekeeping gene hypoxanthine-guanine phosphoribosyltransferase (HPRT) (Figure 1) when compared to RNA prepared from malignant tissue samples. Cells from cell cultures were disrupted and lyzed in RLT buffer by passing through a 20-gauge needle assembled to a 2-ml syringe. Further procedures followed the manufacturer’s instruction (QIAGEN). Total RNA was incubated with 10 U DNaseI (recombinant, RNase-free, Roche Diagnostics, Mannheim, Germany) to remove genomic DNA. DNaseI was inactivated by addition of EDTA and heated for 10 min at 95°C as recommended by the manufacturer. RNA quantity was measured by NanoDrop ND1000 (PeqLab Biotechnologie GmbH, Erlangen, Germany) and quality was determined for probes with sufficient quantity by gel electrophoresis. About 25 ng of each RNA preparation was tested for DNA contamination by control PCR with mixed oligonucleotide primers (MOP). Only RNA preparations negative for PCR products in 3% agarose gel electrophoresis were used for subsequent reverse transcription and all consecutive RetroArray and qRT-PCR experiments. RNA was reverse transcribed into cDNA with SuperScript® II Reverse Transcriptase (Invitrogen, Carlsbad, CA, USA) and pdN_6_ random primer (Roche Diagnostics) according to the manufacturer’s instructions.

### Retrovirus-specific Microarray (RetroArray)

The RetroArray consists of 50 representative HERV RT-derived sequences from 20 major HERV groups. For design of these capture probes databases were screened for RT-related sequences, which were then classified according to the current nomenclature and further subgrouped with respect to their degree of nucleotide similarity. Representative members of each subgroup were selected with special emphasis on full-length proviral genomes and retroviral sequences that have been associated previously in literature with any biological activities and/or human diseases (Table S1).

RetroArray hybridization probe synthesis, labeling by MOP-PCR as well as printing, blocking, hybridization, and post-processing of retrovirus-specific microarrays were performed as described previously [21], [41], [48]. Hybridized microarrays representing triplicates of the same array on the same chip were scanned using an Affymetrix GMS-418 scanner (laser power setting, 100%; gain setting, 50%), and the resulting images (16-bit TIFF files) were subjected to qualitative analysis using ImaGene 4.0 software (BioDiscovery, Inc., Los Angeles, USA). Exclusively arrays showing reproducible hybridization patterns in triplicate subarrays were further evaluated. Densitometric data were used for discrimination of positive signals from background. As described in the study of Frank et al. [48], an arbitrary cut-off value corresponding to twofold background intensity values of the respective chip was used to discriminate between positive and negative signals. This proved to be in good agreement with the optical appearance of raw images when observed on a color-calibrated monitor in a darkened room. To account for the influence of mRNA quality, HERV signals were compared to RNA levels of the gene HPRT, a housekeeping gene showing the most consistent transcript levels in urothelial tissues. Since RetroArray is in principle a qualitative method due to some design limitations (i.e. use of degenerate primers) as discussed *in extenso* [40], [41], [49], HERV groups showing signals above the mentioned cut-off value were evaluated as active regardless of their signal strength. It should be noted that each positive RetroArray signal may represent multiple HERV loci assigned to one subgroup of multicopy HERV elements with sufficient sequence similarity to preclude identification of individual loci.

### Quantification of HERV Transcripts by qRT-PCR

For the amplification of *pol*(RT) sequences, HERV subgroup-specific *pol* primers for HERV-E4-1 (forward primer: 5′-GGTGTCACTACTCAATACAC-3′, reverse primer: 5′- GCAGCCTAGGTCTCTGG-3′; [21]) and HERV-T(S71-TK1) elements (forward primer: 5′-GTACCCCAGGTAGGAAACTCTGGG-3′, reverse primer: 5′-CCCCTACCCTTTTTGGGG-3′; M. Vincendeau, unpublished data), and group-specific degenerate primers for group HERV-K(HML-6) were used [50]. For the locus-specific amplification of HERV-Ec1 in PLA2G4A, provirus-specific *gag* primers were established (forward primer: 5′-GAGTTCCCATATGTAAATTATTGGC-3′, reverse primer, 5′-CTCTTCTGTCTACTCTGTGTGA-3′; chromosome 1∶185157346-185157649). For normalization, the housekeeping genes glycerinaldehyde-3-phosphate-dehydrogenase (GAPDH) (forward primer: 5′-AGTCAACGGATTTGGTCGTATTGGG-3′, reverse primer, 5′-ACGTACTCAGCGCCAGCATCG-3′) and/or glucose-6-phosphate dehydrogenase (G6PD) (forward primer: 5′-TGCAGATGCTGTGTCTGG-3′, reverse primer: 5′-CGTACTGGCCCAGGACC-3′) were amplified. For quantification of PLA2G4A transcript levels, the commercial Hs_PLA2G4A_1_SG QuantiTect Primer Assay (QIAGEN) was employed according to the instructions (two-step Light Cycler 480 protocol) of the manufacturer.

QRT-PCR was performed with the Roche LightCycler 480 System, using LC480 DNA Master SYBR Green and the standard LightCycler protocol (Roche Diagnostics). Two µl of cDNA (an about 50 ng RNA equivalent) were added to 18 µl of reaction mix containing primers at 0.5 µM for the HERV targets and at 0.25 µM for GAPDH or G6PD in LightCycler® FastStart DNA Master^PLUS^ SYBR Green I ready-to-use hot-start PCR mix with *Taq* DNA polymerase (Roche Diagnostics) diluted with purified water according to the manufacturer’s protocol. An initial denaturation step of 10 min at 95°C was followed by 45 amplification cycles of 10 s at 54°C for HERV targets and 60°C for GAPDH or G6PD, and an elongation step of 15 s at 72°C. Melting curves were generated for the final PCR products by decreasing the temperature to 65°C for 15 s followed by an increase in temperature to 95°C. Fluorescence was measured at 0.2°C increments. Housekeeping gene (GAPDH, G6PD) transcripts were analyzed as internal standard. ΔC_T_-values were calculated as follows: C_T_(G6PD)-C_T_(HERV element), and were normalized to G6PD levels. The x-fold induction of HERV transcription in treated cells was calculated by the 2^−ΔΔCT^ method [51], with values normalized to G6PD and relative to transcription in untreated control cells. The relative HERV transcription ratio of samples was calculated from the qRT-PCR efficiencies and the crossing point deviations of the target gene versus G6PD [52]. Quantitative RT-PCR experiments for each gene were performed at least in triplicate.

### Statistics

Statistical analysis was performed with SPSS 15.0. Approximate normal distribution of parametric results was confirmed by linearity in Q-Q plot. Differences between the sample groups were calculated by Student’s *t* test. A *p-*value of <0.05 was considered significant.

Cloning, DNA sequence analysis and genomic mapping of HERV transcripts Aliquots of HERV-E4-1 and HERV-K(HML-6)-specific *pol* qRT-PCR products were purified (QIAquick PCR purification kit, QIAGEN) and cloned into the pCR2.1-TOPO vector (TOPO TA cloning kit, Invitrogen), and E. coli TOP10F’ bacterial cells were chemically transformed with ligation products. Plasmid DNA was isolated from insert-containing colonies according to the manufacturer’s protocol (QuickLyse Miniprep kit, QIAGEN). Subsequently, cloned cDNAs were subjected to custom DNA sequencing (SequiServe, Vaterstetten, Germany). HERV sequences were confirmed by RepBase (www.girinst.org, [19]). For localization of transcribed proviruses in the human genome sequence, we used the BLAT tool at the Human Genome Browser database [53]. Cloned cDNA sequences served as query to search the March 2006 (hg18) version of the human genome. Except for HERV-Ec8A, for which two equivalent loci on chromosome 8 within a duplicated genome region were obtained, assignment of all 40 cDNAs to proviral loci was unambiguous because of more than 6% sequence divergence between the various HERV-E4-1 loci in the human genome. Relative cloning frequencies were used to calculate the relative transcript levels of mapped HERV loci [54].

### Rapid Amplification of cDNA Ends with PCR (RACE-PCR)

For detection and characterization of HERV-Ec1 LTR-driven transcripts we screened DNA-free RNA from UROtsa cells by RACE-PCR (Second Generation 5′/3′-RACE Kit, Roche Diagnostics) according to the manufacturer’s instructions. Protocol and primer design were adapted to identify transcripts preferentially initiated within the 5′- or 3′-LTR of HERV-Ec1. In brief, cDNA first strands (random hexamers) were primed with a biotinylated primer matching the U5 regions of HERV-Ec1 5′- and 3′-LTRs (5′-Biotin-TCAGGGAGCTCGGCTCTTGAGACAG-3′) for complementary second strand cDNA synthesis. Subsequent linear second strand amplification comprised 30 cycles of 96°C denaturation, 67°C annealing temperature and 72°C for elongation. Biotinylated PCR products were separated from the reaction mixture using streptavidin beads M270 according to the instructions of the manufacturer (Invitrogen). Purified second strand cDNA was resuspended in PCR grade H_2_O and tailed using terminal transferase provided with the kit. For subsequent hemi-nested PCR, RACE anchor primers supplied in the kit (Roche Diagnostics) were used in combination with the LTR-specific primers 5′LTR-SP2: CCGATGCTCCCGGCCAAAC or 3′LTR-SP2: AGGAGTCTTGCCGATGCTCCA. For the second amplification step, RACE anchor primers and 5′/3′LTR-SP3: CCTTCCTTCTTTAACTTGGTGTCTGA were used. TOPO TA cloning and DNA sequence analysis were performed as already described.

### HERV-Ec1 siRNA Design and siRNA Treatment of UROtsa Cells

Using the i-Score Designer (http://www.med.nagoya-u.ac.jp/neurogenetics/i_Score/i_score.html) and the s-BioPredSi-Algorithm [55] we designed four specific siRNAs matching the U5 regions of HERV-Ec1 5′- and 3′-LTRs. Custom synthesis of siRNAs (LTR1, target sequence: CCCCAGCTGAATAAAGCCCTT; LTR2, target sequence: AAGCCCTTAAAAGGGACAGAA; LTR3, target sequence: CATAGAGTTGTGAGCCCTTAA; LTR4, target sequence: CTAGTGTTGTGAGCCCTTAAA) was performed by QIAGEN. PLA2G4A-specific siRNA (FlexiTube GeneSolution GS5321) was purchased from QIAGEN. For siRNA treatment, 5×10^5^ UROtsa cells were seeded in triplicates in 6-well dishes (Greiner Bio-One, Frickenhausen, Germany) in 1.5 ml RPMI 1640 (GIBCO/Invitrogen, Karlsruhe, Germany) without supplementation before transfection. After 2 h, cells were transfected with a siRNA cocktail diluted in OptiMEM I (GIBCO) containing 9 pmol of each siRNA using Lipofectamine 2000 reagent following the manufacturer’s instructions (Invitrogen). The resulting final siRNA concentration was about 6 nM per each. Accordingly, 9 pmol of non-targeting control siRNA and MAPK-directed siRNA (QIAGEN) served as controls. After incubation for 5 h at 37°C cell culture medium was replaced by 2 ml fresh RPMI 1640 containing 10% FCS, 2% glutamine and 1% Penicillin-Streptomycin (GIBCO). After 48 h, cells were harvested and total RNA was prepared using the TRIZOL procedure according to the recommendations of the manufacturer (Biozol, Eching, Germany). DNaseI treatment and MOP-PCR controls were performed as described before.

## Results

### HERV Transcription Profiles in Urothelial Carcinoma and Non-malignant Urothelium

To investigate HERV transcriptional activity, we performed RetroArray analysis on 13 pairs of non-malignant (N) and urothelial carcinoma (T) tissue samples, each pair derived from the same patient (Table 1). A digitally processed alignment of a representative raw data set for all patient samples including five urothelial cell lines is shown in Figure 1. The housekeeping gene HPRT served as internal control for RNA quality. Variations in RNA quality from clinical samples and/or the limiting amounts of non-malignant tissues led to weaker or stronger signals for each individual sample. In some cases (patient IDs: 2, 3, 4, 5, 10, 13) cancer-related RNA quality was at large better leading to brighter HERV signatures compared to RNA derived from corresponding non-malignant samples.

**Table 1:** Paired and unpaired patient samples.

Tumor stage*	RetroArray samples (paired)	Additional samples used only forqRT-PCR (unpaired)	qRT-PCR total samples
non-malignant	13	5	18
pTa	5	14	19
pT1	3	2	5
pT2-4/pTis	5	5	10
**Total number**	**26**	**26**	**52**

* Tumor staging according to Grignon [43].

Densitometric normalization to HPRT according to Frank et al. [48] and signal cut-off calculation (data not shown) revealed a distinct urothelium-specific HERV core signature consisting of six active HERV subgroups (HERV-E4-1, HERV-Rb, ERV9, HERV-K-T47D, NMWV3, HERV-KC4) that are transcribed in at least 11 of 13 non-malignant tissue samples (Table 2). This signature is clearly distinct from that found in other tissues [21], [48], [56], [57]. In addition, a variety of differentially active HERVs (observed in 1 to 10 of 13 samples) were identified in non-malignant tissues (Table 2) pointing to variable HERV activity in individual patients. These variations may be explained by a different epigenetic background.

**Table 2:** HERV transcriptional profile of the human urothelium^1^.

HERV group	subgroup (n = 50)	patient non-malignant tissue (n = 13)	patient malignant tissue (n = 13)	normal cell line (n = 1)^2^	cancer cell lines (n = 4)^3^
HERV-I	HERV-I	1	1	0	1
	HERV-IP-T47D	5	7	1	2
	Seq65	5	6	1	3
HERV-T	HERV-T(S71-TK6)	7	7	1	3
	HERV-T(S71-TK1)^4^	3	6	1	3
HERV-FRD	HERV-FRD	5	9	1	4
	HS49C23	0	0	0	0
	HERV-Z	5	7	1	2
HERV-E	HERV-E4-1^4,5^	13	13	1	4
	Seq32	6	10	1	3
HERV-H	RGH2	1	4	1	3
	HERV-H (AF026252)	1	0	0	0
	Seq66	0	0	0	0
HERV-F	HERV-F2	0	0	0	0
	HERV-F	0	3	1	1
	HERV-Fb	10	12	1	4
HERV-W	HERV-W	9	12	1	4
HERV-R	ERV-3	6	7	1	4
	HERV-Rb^5^	11	13	1	3
ERV9	Seq64	4	4	1	2
	Seq63	7	12	1	4
	ERV9^5^	12	12	1	4
	Seq59	10	12	1	4
	Seq60	5	10	1	3
HERV-K(HML-1)	HML-1	7	6	0	2
	Seq29	4	5	0	2
HERV-K(HML-2)	HERV-K10	2	5	1	3
	HERV-K(2.HOM)	1	1	0	0
	HERV-K(HP1)	4	8	1	3
	HERV-K(D1.2)	4	8	1	3
HERV-K(HML-3)	Seq26	3	8	1	3
	Seq34	4	8	1	3
	HML-3	7	10	1	3
	HERV 1	3	4	1	3
	Seq43	1	3	0	0
HERV-K(HML-4)	HERV-K-T47D^5^	11	11	1	4
HERV-K(HML-5)	HML-5	0	0	0	0
HERV-K(HML-6)	HML-6^4^	6	10	1	2
	Seq38	10	11	1	3
	Seq56	8	10	1	4
HERV-K(HML-7)	NMWV7	2	4	0	0
HERV-K(HML-8)	NMWV3^5^	13	13	1	4
HERV-K(HML-9)	NMWV9	7	8	1	4
HERV-K(HML-10)	HERV-KC4^5^	11	12	1	4
	Seq31	6	7	1	4
HERV-L	HERV-L	5	8	1	3
	Seq39	4	5	1	3
	Seq45	5	6	1	3
	Seq51	5	8	1	3
	Seq58	0	0	0	0

1 Numbers of samples positive for the respective HERV out of 13 paired (malignant and non-malignant) patient samples. Samples were considered positive, when their corresponding signals exceeded the calculated cut-off value as described in Materials & Methods. Incidences are given by absolute numbers of samples and do not represent any quantitative assessment of HERV transcript levels.

2 UROtsa cells were used as model system of the normal human urothelium.

3 Tumor cell lines under investigation: RT112, T24/83, UMUC-3, HT1197.

4 HERV subgroups selected for quantification by qRT-PCR.

5 HERV subgroups that represent the urothelium-specific core HERV signature.

In order to search for cancer-related HERV activity, we compared the signatures of pairs of non-malignant and malignant tissue samples (Figure 1 and Table 2). None of the HERV subgroups inactive for *pol* transcription in non-malignant urothelium became transcriptionally activated in the corresponding urothelial carcinoma. A comparison of HERV incidences revealed that the signatures of malignant samples were generally similar to those of non-malignant tissue samples. This holds true for the non-malignant (UROtsa) and tumor cell lines (Rt112, T24/83, UMUC-3, Ht1197) included in the study. As shown in Figure 1, the same HERV subgroups (HERV-E4-1, HERV-Rb, ERV9, HERV-K-T47D, NMWV3, HERV-KC4) that make up the characteristic core signature are equally represented in non-malignant and malignant tissues. A similar distribution (no significant changes in incidences) was also observed for the variably transcribed HERVs underlining the importance of pairwise comparisons. However, it should be noted that each signaling spot on the microarray detects transcripts from multiple HERV loci of a subgroup of multicopy HERV elements, so that single loci cannot be distinguished. Therefore, activation of individual HERV loci in cancer tissue may be masked by an overall transcription of members of the same HERV subgroups equally transcribed in normal and tumorous tissue.

### Quantification of HERV-E4-1, HERV-K(HML-6) and HERV-T(S71-TK1) Transcript Levels

To examine a potential increase or decrease of HERV transcript levels in tumorous tissue compared to non-malignant tissue, we selected three expressed HERV subgroups, HERV-E4-1, HERV-K(HML-6) and HERV-T(S71-TK1) for qRT-PCR. Based on our own previous data [56], and on reports of others [35], [58], [59], members of HERV-E4-1 and HERV-K(HML-6) have been implicated in various human cancers. HERV-T(S71-TK1), to date not connected with any known disease, was chosen for reasons of comparison.

**Figure 2:**
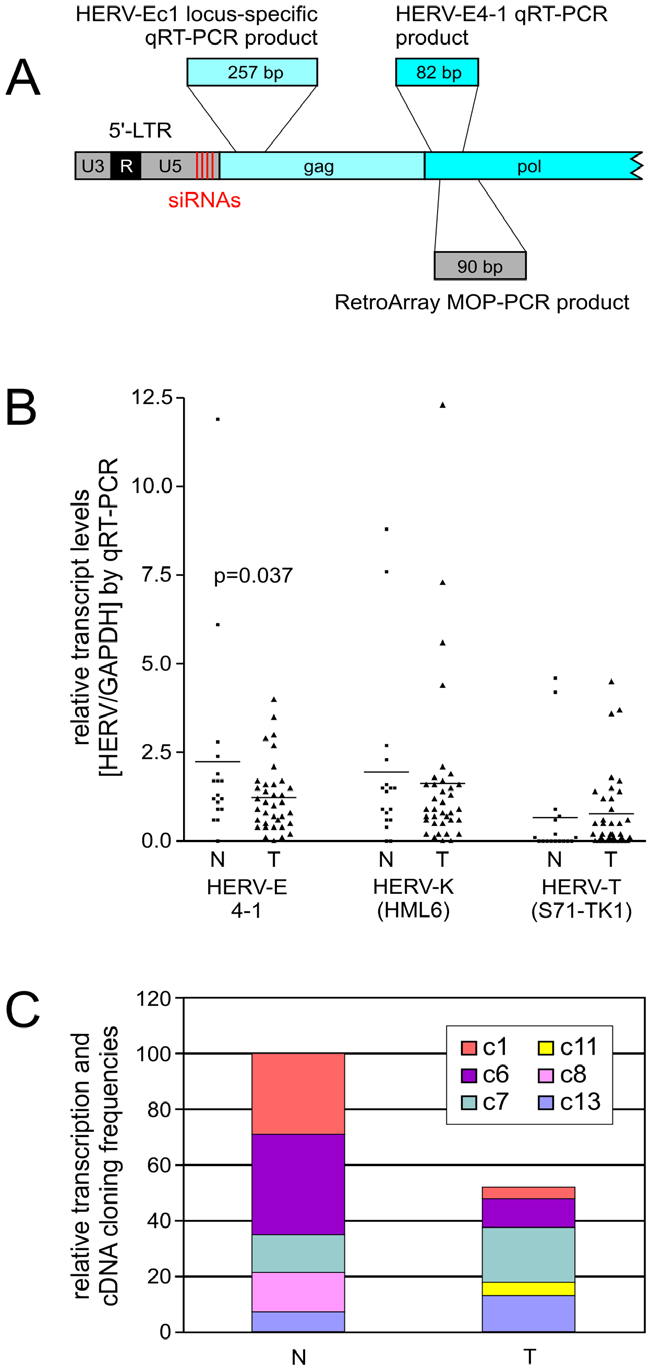
Relative transcript levels of selected HERV elements in patient samples. (A) Relative positions (not drawn to scale) of siRNAs (red bars) in the HERV-E4-1 5′-LTR. Location and sizes of qRT-PCR and MOP-PCR products (RetroArray) are shown. Given amplicon sizes exclude PCR primers. (B) Quantitative analysis of HERV-E4-1, HERV-K(HML-6) and HERV-T(S71-TK1) transcript levels in cDNA samples derived from 18 non-malignant urothelium (N) and 35 urothelial carcinoma (T) tissue specimen, including 16 paired tissue samples. HERV subgroup-specific *pol* primers for HERV-E4-1 and HERV-T(S71-TK1), and degenerated *pol* primers for HERV-K(HML-6) were used. Relative transcript levels were quantified according to Pfaffl [52]. All qRT-PCR values were normalized to GAPDH levels. HERV-E4-1 mean expression of N vs. T was significantly different (p = 0.037; Student’s *t*-test). (C) Relative cloning frequencies of HERV-E4-1-related cDNAs (as shown in Tab. 3) were combined with the respective HERV-E4-1 qRT-PCR data (Figure 2B) to illustrate the differential activities of transcriptionally active HERV-E4-1 loci in malignant (T) and non-malignant (N) tissues of patient no. 2.

QRT-PCR was performed on all paired tissue samples available (n = 26) and additionally on 26 unpaired samples (5 non-malignant, 21 malignant; Table 1). We employed HERV subgroup-specific *pol* primers located juxtaposed to the MOP used for RetroArray analysis (see Figure 2A for localization of HERV-E4-1 primers). As shown in Figure 2B, the HERV-E4-1 subgroup displays reduction to 48% (p = 0.037) in transcript levels whereas HERV-K(HML-6) and HERV-T(S71-TK1) show no significant alteration in malignant compared to non-malignant tissue samples.

### Identification of Transcribed HERV-E4-1 Loci

To investigate the possibility of differential activity of single HERV-E4-1 loci not detectable by the microarray or subgroup specific qRT-PCR we cloned and sequenced the qRT-PCR amplicons of a patient with urothelial carcinoma stage pT1 (patient no. 2 in Figure 1). This patient showed a lower HERV-E4-1 transcript level in tumor tissue. In total, 40 individual cDNA clones for HERV-E4-1 were analyzed. The cDNA sequences, 82 base pairs (bp) in length when excluding PCR primers, displayed 96% to 100% sequence identity with the respective provirus, sufficient for unambiguous assignment and thus identification of transcribed HERV loci (Figure S1). Genomic loci and relative abundance of transcripts according to cloning frequencies of corresponding cDNAs is shown in Table 3. Five HERV-E4-1 loci (HERV-Ec1, -Ec6, -Ec7, -Ec11, -Ec13) and one ambiguous locus (HERV-Ec8) were identified. The latter provirus is located within an intrachromosomally duplicated genome region and both HERV-Ec8 loci differ by only 20 nt per 8 kb proviral sequence making assignment to one or the other locus impossible. The HERV-E4-1 elements located on 1q31.1, 6q15, 7q21.3, and 13q14.1 were transcribed in both normal and urothelial carcinoma tissue samples. HERV-Ec8 transcripts were only found in non-malignant tissue, HERV-Ec11 only in malignant tissue of the analyzed patient. While the levels of HERV-Ec1 and HERV-Ec6 transcripts were decreased in malignant tissue, the opposite effect was observed for transcript HERV-Ec13. Transcripts derived from locus 8p23 are identical with a HERV-E4-1 sequence described by Prusty and coworkers who localized on 8p23 a HERV-E4-1 provirus expressing an almost full length transcript of 7.1 kb in leukemic and hematopoietic cells [58]. The itemization of active HERV-E4-1 loci with respect to their contribution to total transcript levels observed in malignant and non-malignant tissues by qRT-PCR revealed a reduction of HERV-Ec1 and HERV-Ec6 transcription in malignant tissue. Minor increases were observed for HERV-Ec7, HERV-Ec11 and HERV-Ec13 whereas HERV-Ec8 was not detected in malignant tissue (Figure 2C, Table 3).

**Table 3:** Chromosomal localization and relative cloning frequencies of cDNAs derived from transcribed HERV-E4-1 loci in malignant and non-malignant tissue of patient no. 2.

Transcript^1^	Location of amplicon(proviral locus)^2^	Chrom. localization	cloning frequency in non-malignant tissue (%)	cloning frequency in malignant tissue (%)	Proximity to genes (<100 kb distance)	found in tissue,reference
HERV-Ec1	chr1∶185155301–185155383	1q31.1	29%	7%	PLA2G4A, withinintron 7	–
HERV-Ec6 (ERVE-4)	chr6∶89432125–89432208	6q15	36%	20%	RNGTT, withinintron 14	thalamus (Genbank; AK125006)
HERV-Ec7	chr7∶97420616–97420698	7q21.3	14%	40%	ASNA (5′, 81 kb) OCM2 (3′, 31 kb)	–
HERV-Ec8 (ERVE-3)	chr8∶8070254–8070336 or chr8∶12392139–12392221^3^	8p23.1 or8p23.1	14%	0%	–	leukemic cells (Prusty et al. [58])
HERV-Ec11	chr11∶3456532–3456614	11p15.4	0%	7%	ZNF195 (5′, 99 kb)	–
HERV-Ec13	chr13∶40349963–40350045	13q14.1	7%	26%	SLC25A15 (5′, 68 kb) SUGT1L1 (3′, 34 kb)	–
			100% (14)^4^	100% (15)^4^		

1 aliases according to HUGO Gene Nomenclature Committee are in parentheses [90].

2 Chromosomal localization of MOP-PCR amplicons according to the hg18/March 2006 human reference genome sequence as given by the UCSC Genome Browser.

3 HERV-Ec8 was mapped within a duplicated genome region, with both HERV-Ec8 sequences displaying only 20 nt differences along 8 kb. Assignment to one or the other locus is therefore not possible due to high sequence similarity.

4 Total number of sequenced cDNA clones.

Abbreviations: no., number; chr, chromosome.

**Figure 3:**
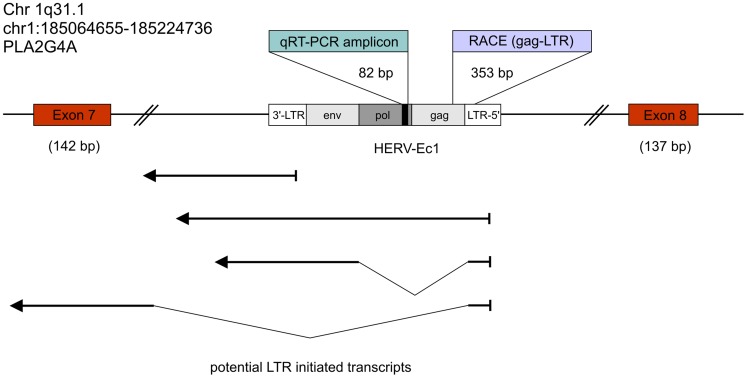
Schematic representation of the PLA2G4A gene locus harboring an intronic HERV-E4-1 element in antisense orientation. Genomic location (not drawn to scale) of HERV-E4-1 located in the intron of PLA2G4A. The qRT-PCR amplicon (82 bp in length) also represents the cDNA sequence for identifying transcribed HERV-E4-1 loci by using BLAT on the hg18 human reference genome at the UCSC Genome Browser [53]. A 5′-LTR RACE product with a length of 353 bp was detected as described in Materials & Methods. Potential HERV-E4-1 LTR-initiated transcripts are given by bold arrow lines. Potential splicing is depicted by thin lines.

Two of the transcribed HERV-E4-1 loci, HERV-Ec1 and HERV-Ec6, were found within gene introns, both in antisense orientation. The other HERV-E4-1 proviruses are located in intergenic regions. The distances of HERV-Ec7, HERV-Ec8, HERV-Ec11 and HERV-Ec13 to proximate genes range between 31 kb and several hundred kb (Table 3). HERV-Ec1 is located between exon 7 and 8 of the PLA2G4A gene and HERV-Ec6 between exon 14 and 15 of the RNGTT (RNA guanylyltransferase and 5′-phosphatase) gene. As outlined in Figure 3 for PLA2G4A, both HERV-E4-1 loci represent full length proviruses (ca. 8.8 kb) with 5′- and 3′-LTR sequences. According to the observed cloning frequencies, both HERV-E4-1 loci are transcribed at lower levels in malignant tissue than in non-malignant tissue (Table 3, Figure 2C). Since numerous reports on dysregulation of PLA2G4A in a variety of cancers point to a possible role of this gene in tumor growth, tumor cell migration and tumor angiogenesis (reviewed in [60]), we focused on the HERV-Ec1 provirus and its potential role in regulation of the PLA2G4A gene.

### Antagonistic Regulation of PLA2G4A and HERV-Ec1 Transcript Levels

**Figure 4:**
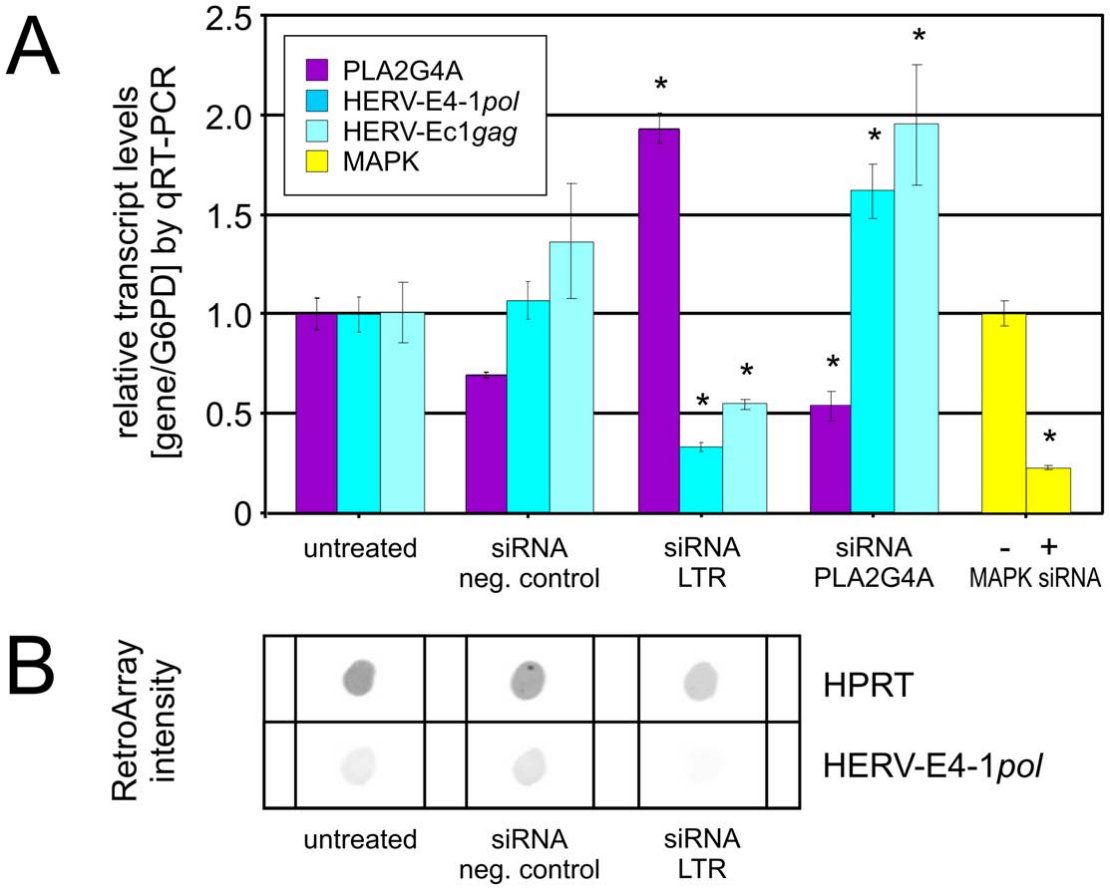
Relative transcript levels of HERV-E4-1 and PLA2G4A in UROtsa cells after siRNA treatment. Cells were treated with negative control siRNA, HERV-E-LTR-specific siRNA (pool of 4 different siRNAs), and PLA2G4A-specific siRNA. As controls, untreated cells and cells transfected with MAPK-directed siRNA (+ control) were used. (A) QRT-PCR assays were performed on DNA-free RNA samples that were obtained from three independent siRNA transfection experiments. For assessment of HERV-E4-1 transcript levels primers derived from the *pol* and *gag* region were used. Transcripts of MAPK and PLA2G4A were analyzed using gene specific primers. While HERV-E4-1 *pol*-targeting primers overlap with the capture probe of the RetroArray and may amplify several HERV-E4-1 loci, the HERV-Ec1 *gag* primers are specific for the HERV-Ec1 provirus located in the PLA2G4A gene. Relative transcript levels were normalized to G6PD (housekeeping gene) levels and represent the mean value of at least triplicate qRT-PCR assays. Numbers on the Y-axis show the fold change of transcription. P-values as shown in Figure 4A are given in order from left to right (p = 0.0015, p = 0.009, p = 0.044, p = 0.04, p = 0.0005, p = 0.018, p = 0.0014). (B) RetroArray signal intensity of the same samples showing HPRT (housekeeping gene) and overall HERV-E4-1*pol* transcript levels.

To investigate potential regulatory effects of HERV-Ec1 transcription on PLA2G4A expression, we performed siRNA experiments in UROtsa cells that served as a model system for the non-malignant human urothelium [41], [45]. Transcripts of PLA2G4A were detected in untreated UROtsa cells at levels comparable to those of common housekeeping genes GAPDH and G6PD (data not shown). We transfected UROtsa cells with four siRNAs targeting the U5 region of HERV-Ec1 5′- and 3′-LTR and a siRNA against transcripts of the PLA2G4A gene. After incubation for 48 h, PLA2G4A, HERV-E4-1-*pol* and HERV-Ec1-*gag* transcription was measured by qRT-PCR. Whereas the HERV-E4-1-*pol* primers are subgroup-specific and amplify virtually all elements of the HERV-E subgroup E4-1, HERV-Ec1-*gag* primers are strictly locus-specific, as was demonstrated by cloning and DNA sequencing of the amplicon (data not shown). Compared to untreated control cells, siRNA treatment increased PLA2G4A transcript levels twofold (1.93+/−0.08) (Figure 4A) while HERV-E4-1-*pol* and HERV-Ec1-*gag* transcript levels were reduced to 0.31+/−0.02 and 0.55+/−0.03, respectively. On the contrary, PLA2G4A-specific siRNA treatment reduced PLA2G4A transcript levels (0.55+/−0.11) and increased HERV-E4-1-*pol* (1.64+/−0.17) and HERV-Ec1-*gag* (1.95+/−0.31) transcript levels. Downregulation of the HERV-E4-1 subgroup by siRNA treatment was further confirmed by RetroArray analysis (Figure 4B). Thus, downregulation of HERV-Ec1 transcription concurred with PLA2G4A upregulation (p = 0.0015).

**Figure 5:**
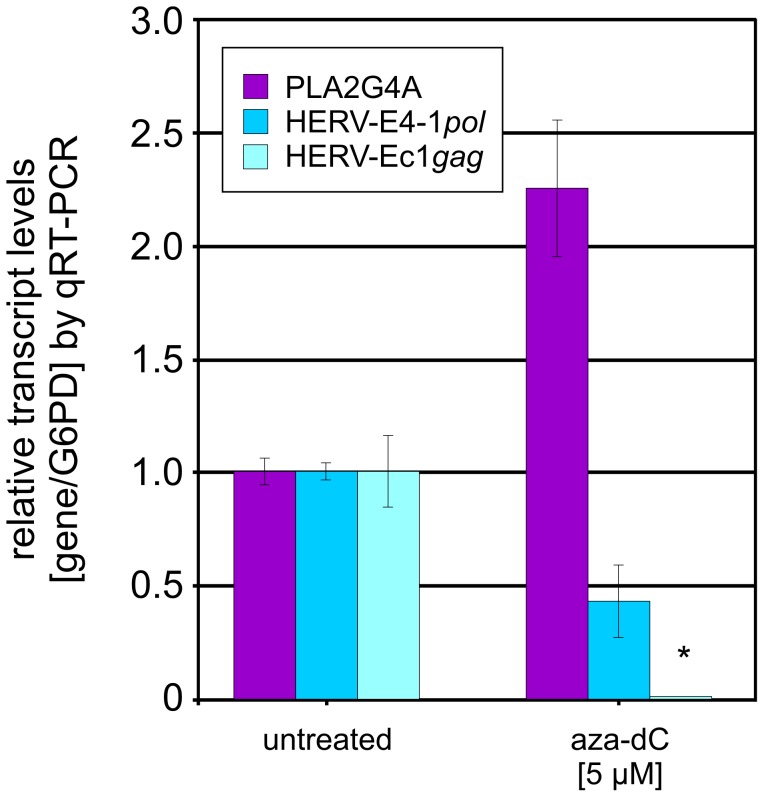
Effect of aza-dC on transcription of PLA2G4A in UROtsa cells. UROtsa cells were incubated with 5 µM 5-aza-2-deoxycytidine (aza-dC) for 48 h and analyzed by qRT-PCR in comparison to untreated cells. QRT-PCR assays were performed on DNA-free RNA samples derived from two independent experiments. Primer pairs derived from *pol* and *gag* regions were used. While HERV-E4-1 *pol*-targeting primers overlap the capture probes of the RetroArray and may amplify several HERV-E4-1 loci, the HERV-Ec1 *gag* primers are specific for the HERV-Ec1 provirus located within intron 7 of the PLA2G4A gene. Transcript levels of PLA2G4A were analyzed using gene-specific primers. Relative transcription levels were normalized to G6PD levels and represent the mean value of six qRT-PCR assays. Numbers on the Y-axis show the fold change of transcription. (*) An almost complete downregulation of HERV-Ec1 *gag* transcription (0.00027±0.00017%) was observed.

In a second approach we treated UROtsa cells with the DNA methylation inhibitor aza-dC in an analogous experimental setting. In this setting, the PLA2G4A promoter as well as the HERV promoter should be activated. UROtsa cells were incubated for 48 h with subtoxic doses of aza-dC and the relative transcript levels of targets were determined by qRT-PCR (Figure 5). Compared to untreated cells, aza-dC treatment of UROtsa cells resulted in an increase of PLA2G4A transcripts (2.24+/−0.29) and an almost complete downregulation (0.0003+/−0.0002) of the corresponding HERV-Ec1-*gag* transcript levels. This suggests a mutual regulatory dependency between PLA2G4A and its intron HERV-E4-1 element, whereby the PLA2G4A promoter exhibits the stronger activity.

**Figure 6:**
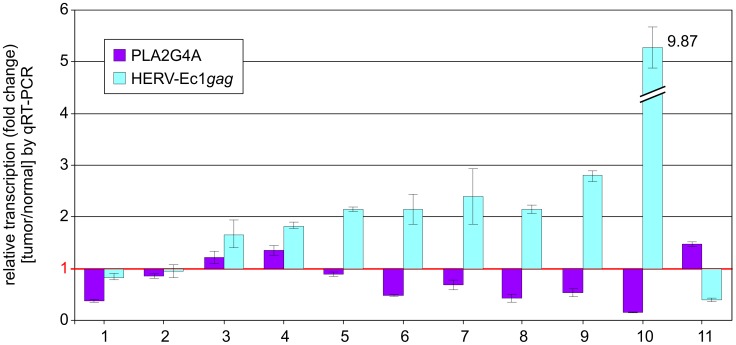
Comparative analysis of HERV-Ec1 *gag* and PLA2G4A transcription in patients with urothelial carcinoma. QRT-PCR assays were performed on DNA-free RNA samples obtained from patients with urothelial carcinoma (n = 11). Due to lack of material, additional patients were used not shown in Figures 1 and 2. For measurement of HERV-Ec1 transcripts locus-specific primers derived from the *gag* region of HERV-Ec1 were utilized. Transcript levels of PLA2G4A were analyzed using a gene specific primer set. Relative transcription levels were normalized to G6PD transcript levels and represent the mean value of triplicate qRT-PCR assays. The numbers on the Y-axis show the fold change of transcription in urothelial carcinoma with respect to corresponding mean value of non-malignant tissue samples (value = 1, depicted by red horizontal line). Thus, values >1 denote increases, values <1 correspond to decreases in transcript levels.

To compare the data obtained from UROtsa cells with the situation *in vivo*, we analyzed PLA2G4A and HERV-Ec1-*gag* transcript levels in DNA-free RNA preparations from additional patients (n = 11) with urothelial carcinoma. Paired non-malignant and malignant tissue samples were examined by PLA2G4A and HERV-Ec1-*gag* qRT-PCR. As shown in Figure 6, patient samples can be divided in three groups. In group I (patients 1–4), changes in relative transcript levels below 2-fold were observed. In all cases, both transcripts were either reduced or elevated and no reciprocal relation was observed. Group II comprising patients (5–10) shows a reciprocal transcript pattern. HERV-Ec1-*gag* transcript levels were found upregulated, while PLA2G4A levels were downregulated. Patient 11 showed a reversed expression pattern with increased levels of PLA2G4A and decreased levels of the HERV-Ec1 transcript. Since seven of eleven patients showed a reciprocal transcript pattern for PLA2G4A and HERV-Ec1-*gag* transcripts *in vivo* confirming our *in vitro* data, we suggest that HERV-Ec1 located in intron 7 of PLA2G4A may contribute to fine tuning of the PLA2G4A gene expression. No correlation between tumor stage and transcript pattern was found.

To further characterize the nature of the HERV-Ec1-initiated (antisense) transcript and to discriminate between potential 5′- or 3′-LTR-initiated HERV-Ec1 transcription, we performed reverse transcriptase PCR and RACE-PCR on DNA-free preparations of UROtsa RNA. Combinations of various primers derived from the U5 region of HERV-Ec1 LTRs (forward strand) and from PLA2G4A Exon 4–7 and intronic regions of PLA2G4A gene (all from the reverse strand) were used for amplification. While positive control PCR with UROtsa DNA as template amplified the expected short LTR-PLA2G4A amplicons, cDNA as template failed to amplify LTR-PLA2G4A-derived chimeric products (data not shown). RACE-PCR yielded products of up to 353 bp in length, all derived from the 5′-LTR and the adjacent *gag* gene of HERV-Ec1 confirming transcriptional activity of the 5′-LTR. No transcripts initiating from the 3′-LTR or read-through transcripts starting from the 5′-LTR and going beyond the 3′-LTR of HERV-Ec1 were detected (data not shown). To exclude the possibility of a qRT-PCR bias due to presence of unspliced pre-mRNA of the PLA2G4A gene in our RNA preparation, we performed PCR with HERV-Ec1 LTR-specific primers matching the opposite DNA strand and intron-specific primers located 5′ of the HERV-Ec1 provirus. No PCR products were observed suggesting the lack of unspliced PLA2G4A pre-mRNA containing the intronic HERV-Ec1 provirus in our PCR template preparation.

## Discussion

### The HERV Transcription Profile is Largely Identical in Human Urothelial Carcinoma, Non-malignant Urothelial Tissue, Four Tumor Derived Cell Lines and in a Non-malignant Urothelial Cell Line

Using RetroArray we have established the HERV expression landscape of the human urothelium. The core HERV transcription profile of urothelial tissue consists of six HERV subgroups including class I (HERV-E4-1, HERV-Rb, ERV-9) and class II (HERV-K-T47D, NMWV3, HERV-KC4) elements. Comparison with HERV core patterns (Table S2) previously established for human brain, mammary gland and kidney confirmed two ubiquitously active HERV subgroups (HERV-E4-1, ERV-9) that are part of the core signature observed in all three tissues [48], [56], [57]. HERV-K-T47D, NMWV3 and HERV-KC4 subgroups are variably expressed in other tissues whereas HERV-Rb transcription was exclusively found in the urothelium.

This supports our earlier findings that HERV elements are active in human cells in a tissue-specific manner [21], [48], [56], [57]. The same HERV transcription profile was found in the non-malignant urothelial cell line UROtsa indicating the cell line’s suitability as a model for subsequent studies on HERV activity in the human urothelium. This is important, because observations of Haupt and coworkers demonstrated expression of additional HERVs when kidney tissue cells from patients are kept in cell culture [57]. In addition to the ubiquitously detected core pattern, a variety of differentially active HERV subgroups of all three HERV classes was found (Figure 1, Table 2). This interindividual transcriptional variability seems to be a hallmark of HERV expression in human tissues and was described previously for human mammary gland, brain and kidney [48], [56], [57]. It may reflect variations of the individual epigenetic background such as DNA methylation pattern and chromatin modifications (O. Diem, unpublished data).

Deregulation of HERV expression has been associated with many cancers (reviewed in [30], [31]). Although RetroArray proved to be a useful tool to detect differential HERV expression in healthy and tumorous tissue [48] we observed no significant differences in HERV incidences between non-malignant and malignant urothelial samples, as well as between UROtsa cells and four tumor cell lines. Regarding the variably active HERV subgroups no significant alterations of the transcription pattern coincide with the malignancy as well (Figure 1, Table 2). Similar observations have been made previously for renal cell carcinoma [57]. No tumor cell line or tumor specific differences were found, suggesting that HERV transcription is not altered in kidney cancer. However, it should be noted here that the RetroArray is based on amplification and amplicon hybridization of a 90 bp sequence located within the *pol* gene [21], [40]. Discrimination of distinctive HERV subgroups is achieved by the use of subgroup-specific *pol* sequences spotted on the chip that are flanked by the highly conserved motifs used as primers for amplification [40]. Therefore, each signaling spot on the microarray may represent transcripts from several closely related HERV proviruses that cannot be distinguished and hybridization signals on the RetroArray provide merely average transcript levels of a given multicopy HERV subgroup.

### Quantification of Three HERV Subgroups, HERV-E4-1, HERV-K(HML-6) and HERV-T(S71-TK1) Reveals a Bias to Lower HERV Activity in Tumor Cells

In further experiments we focused on three HERV subgroups, HERV-E4-1, HERV-K(HML-6) and HERV-T(S71-TK1). HERV-E4-1 elements are part of the core transcription pattern in urothelial tissue, whereas HERV-K(HML-6) sequences and HERV-T(S71-TK1), a subgroup of HERV-T, are variably expressed. HERV-E and HERV-K(HML-6) subgroups were selected because of their history as cancer-associated elements. A HERV-E element has been described to encode a tumor-specific antigen in renal cell carcinoma that may be used for immunotherapy [35]. An increased transcriptional activity of several HERV-K(HML-6) proviral loci was detected in mammary carcinoma [56]. QRT-PCR on all paired and unpaired patient samples (n = 52) revealed a reduction of HERV-E4-1 transcript levels to about 50% (p = 0.037) in urothelial carcinoma, whereas no significant alteration in transcriptional activity was observed for HERV-K(HML-6) and HERV-T(S71-TK1). This is in accordance with previous observations in mammary tumors, where a general tendency to lower overall HERV activity in tumorous tissue combined with increased transcript levels of some specific HERV subgroups in a subset of patients was found [56].

Proviral LTRs are embedded in the transcriptional network of cells. They carry essentially similar regulatory sequences as cellular promoters and therefore obey the same basal rules of the transcriptional machinery of the host cell. Malignant transformation, phenotypically apparent by alterations in proliferation, differentiation, and cell-cell interaction is associated with changes in gene expression, and variations in the transcript level of distinct HERV elements may reflect selective epigenetic alterations of their genomic region [61], [62]. Transcriptional changes of a multitude of cellular genes, including up- and downregulation of transcription factors and other regulatory proteins have been monitored in different stages of urothelial carcinoma development [63], [64], reviewed in [65]. Therefore, differential HERV regulation may reflect changes in the expression network in urothelial carcinoma cells and may result from the malignant transformation, but do not necessarily point to HERVs as etiological agents of malignancy.

### Single HERV-E4-1 Proviral Loci are Differentially Regulated in Urothelial Carcinoma Cells

These considerations were confirmed by analysis of transcribed HERV-E4-1 loci in a paired sample of malignant and non-malignant tissue demonstrating that against the tendency to an overall lower HERV activity in tumors certain proviral loci may be activated or up-regulated. A total of six different HERV-E4-1 transcripts has been detected in malignant and non-malignant urothelial tissue and assigned to specific chromosomal loci; one (HERV-Ec11) was found only in urothelial carcinoma and one (HERV-Ec8) only in non-malignant urothelial tissue. Another HERV-E4-1 cDNA (HERV-Ec8) identified in non-malignant urothelium is identical with a HERV-E transcript detected previously in normal human blood cells and in the chronic myeloid leukemia cell line K562 [58].

Tumor-related expression of group HERV-E sequences has been reported in several previous studies (reviewed in [31]). So far, HERV-E transcripts have been found in breast cancer, ovarian/endometrial cancer, in lung, colon and prostate carcinomas [56], [66], [67], [68], [69], [70], in multiple benign tissues [21], [29], and in multiple normal and malignant cell lines derived from prostate, testis, kidney, and thymus [71]. However, in most cases the corresponding proviruses have not been identified. Two aberrantly spliced HERV-E4-1 transcripts, CT-RCC-8 and -9 that were found to be activated in renal cell carcinoma could be assigned to a provirus located on the long arm of chromosome 6. However, this locus is not transcribed in non-malignant or urothelial carcinoma tissue.

### Mutual Modulation of PLA2G4A and HERV-Ec1 Transcript Levels in Human Urothelial Cells

The search for adjacent cellular genes revealed that four out of six proviruses active in urothelial carcinoma and/or non-malignant urothelial tissue are located in a distance of 30–230 kb 5′ or 3′ to the next gene. Two of the transcribed HERV-E4-1 loci (HERV-Ec1 and HERV-Ec6), are situated within introns of the genes PLA2G4A and RNGTT, both in antisense orientation. According to various studies, LTRs/HERVs in introns can contribute alternative promoters, splicing and polyadenylation signals. This may result in altered/truncated proteins with varying biological functions [4], [5], [72], [73], [74], [75], [76], [77], [78]. Therefore, a significant bias to antisense orientation of HERVs in introns is observed indicating a strong negative selection pressure [79], [80]. However, some HERV LTRs have been shown to exert bidirectional promoter activity and may thus initiate gene transcription in opposite direction [77], [81], [82]. Interestingly, LTR-provided start sites of potential antisense transcripts are more abundant close to the 3′-end of genes and the involved HERV elements are relatively ancient suggesting conservation of their function throughout evolution due to biologically significant regulatory activities [13]. Recently, first evidence was provided for human-specific antisense regulation of gene expression due to LTR promoter activity from intronic HERV sequences [24]. HERV-K(HML-2)-initiated transcripts that are complementary to the mRNAs of genes SLC4A8 and IFT172 were found to reduce the mRNA levels of these genes *in vivo*.

Therefore, we have analyzed the potential effect of the intronic HERV-Ec1 provirus on PLA2G4A transcript levels in UROtsa cells. This cell line shows about the same levels of HERV-Ec1 and PLA2G4A transcripts as human tissue samples. Reciprocal shifts of PLA2G4A and HERV-Ec1 transcript levels after siRNA and aza-dC treatment of UROtsa cells as well as in 7 of 11 patients point toward mutual transcriptional regulation of PLA2G4A and HERV-Ec1 transcription.

As potential regulatory mechanism involving oppositely oriented HERV LTRs in introns, RNA interference including short interfering RNAs or microRNAs is conceivable [13], [83]. Since RACE-PCR detected only 5′-LTR-initiated HERV-Ec1 transcripts, an involvement of the 3′-LTR promoter resulting in read-through transcripts with PLA2G4A intronic and/or exonic upstream sequences is implausible. This is confirmed by transient transfection assay with luciferase as reporter gene pointing to a very low promoter activity of the 3′-LTR (data not shown). Furthermore, no transcripts initiating in the 5′-LTR and splicing from the retroviral splice donor site into exonic or intronic sequences of the PLA2G4A gene could be observed. However, read-through transcripts initiating within the 5′-LTR may use a cryptic splice site within the 3′-flanking sequences to splice into PLA2G4A exons or introns, a mechanism that has been shown previously for ERV3 initiated transcripts of the PLK gene [84]. Because of the restricted amplicon length, such transcripts could not be identified by RACE-PCR.

Alternatively, transcription of PLA2G4A and HERV-Ec1 at the same time may generate competing assemblies of transcriptional complexes on opposite coding DNA strands that lead to collisions between RNA polymerase II complexes tracking along opposite strands of the DNA. This weakens the chance for RNA polymerase II to finish transcription of both genes properly [85], [86]. Selective activation/inhibition of the PLA2G4A or HERV-Ec1 promoter may then lead to excess/deficit of either PLA2G4A or HERV-Ec1 transcripts as has been shown by siRNA and aza-dC treatment of UROtsa cells.

PLA2G4A belongs to the group of cytosolic phospholipase A2 (cPLA2) proteins and catalyzes the release of arachidonic acid from membrane phospholipids. Arachidonic acid is an essential metabolite for eicosanoid production involved in normal cellular homeostasis, inflammation, proliferation, and apoptosis. In tumor cells, cPLA2 transcription and post-translational processing is often dysregulated (reviewed in [60], [87]). In most cases cPLA2 is upregulated; for example, in human lung tumor cells cPLA2 activation by oncogenic Ras has been reported [88]. In contrast, transcriptional suppression of cPLA2 was observed in a subset of colon cancers [89]. It was suggested that downregulation of cPLA2 may attenuate TNF-α mediated apoptosis and thus may facilitate tumor progression. This is in accordance with our observation that in the majority of patients with urothelial carcinoma PLA2G4A is downregulated in the tumor.

The regulation of PLA2G4A gene expression is complex, involving both direct transcriptional activation as well as post-translational modifications. The promoter region of PLA2G4A contains multiple binding sites for various nuclear factor proteins (e.g. Sp1, c-jun) that are known as strong, tissue-specific regulators of gene transcription [60]. Because we observed only low alterations of transcript levels in our *in vitro* experiments, we suppose that the observed regulatory effect of HERV-Ec1 may play a minor role in cPLA2 expression. The biological relevance of the observed reciprocal transcript levels remains elusive, even though in the majority of patients, we observed upregulation of HERV-Ec1 and concomitant downregulation of cPLA2 transcript levels, whereas only one patient (patient 11, see Figure 6) showed an inverse relationship.

In conclusion, we have established a consistent HERV signature for the human urothelium that comprises elements from six transcribed HERV subgroups. Furthermore, a HERV-E-related provirus (HERV-Ec1) transcribed in urothelial carcinoma and non-malignant urothelial tissue was identified that is located in antisense orientation in an intron of the PLA2G4A gene. Transcription of HERV-Ec1 potentially contributes to fine tuning of cPLA2 expression thereby facilitating tumorigenesis.

## Supporting Information

Figure S1 **Sequence divergence among HERV-E4-1 sequences.**
(TIF)

Table S1 **HERV** ***pol*** **sequences used as capture probes for RetroArray.**
(DOC)

Table S2 **Comparison of HERV core transcription pattern in human tissues.**
(DOC)
