# Supplementary figures and images for: HERV-E-Mediated Modulation of PLA2G4A Transcription in Urothelial Carcinoma
Source: PLoS One. 2012 Nov 7;7(11):e49341. doi: 10.1371/journal.pone.0049341 (PMC3492278; doi:10.1371/journal.pone.0049341)

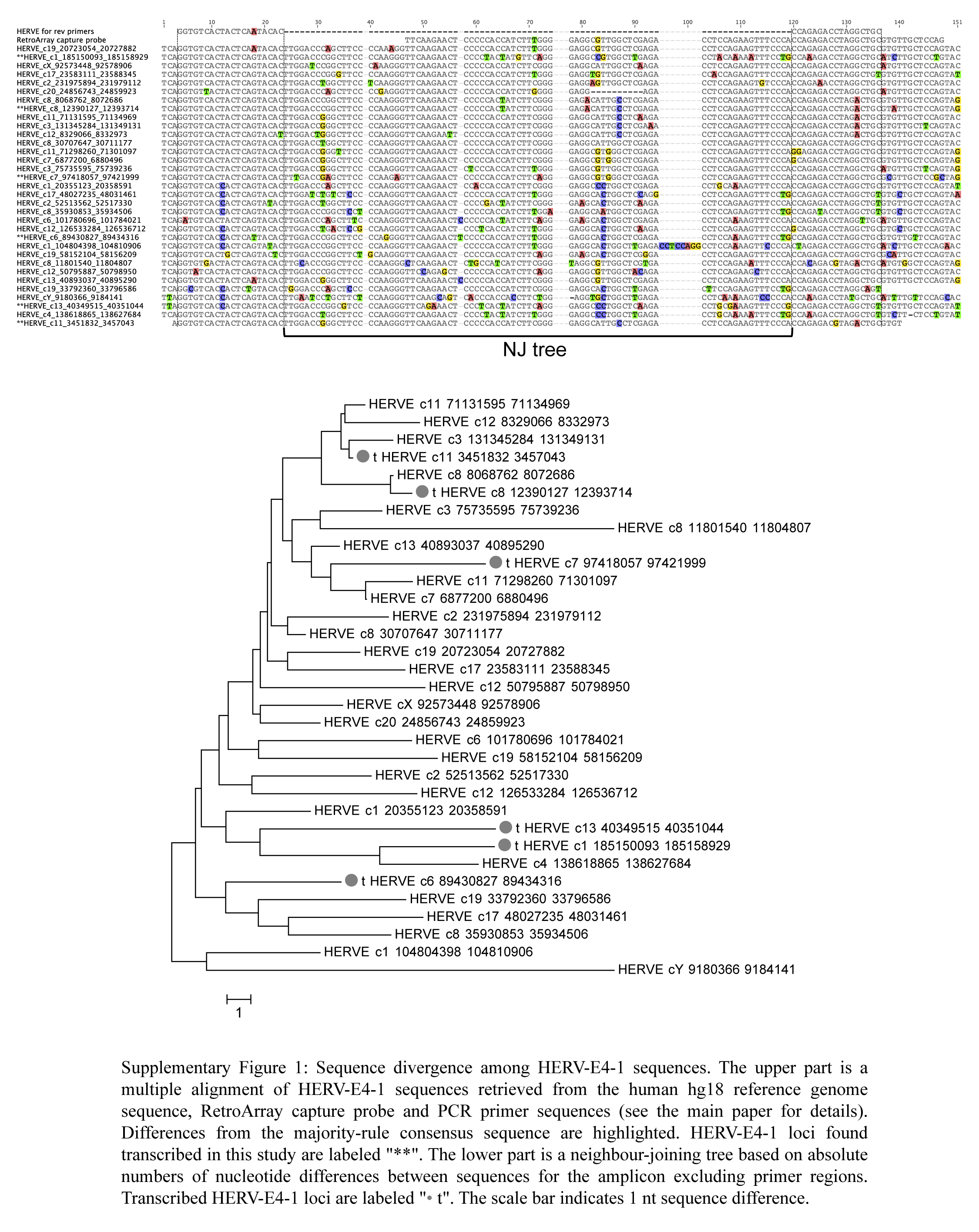

Supplement: Figure S1 — Sequence divergence among HERV-E4-1 sequences. (TIF) [file pone.0049341.s001.tif]
