# Supplementary material for: HERV-E-Mediated Modulation of PLA2G4A Transcription in Urothelial Carcinoma
Source: PLoS One. 2012 Nov 7;7(11):e49341. doi: 10.1371/journal.pone.0049341 (PMC3492278; doi:10.1371/journal.pone.0049341)
Supplement: Table S1 — HERV pol sequences used as capture probes for RetroArray. (DOC) [file pone.0049341.s002.doc]

**Table S1.** HERV *pol* sequences used as capture probes for RetroArray

| HERV class | HERV group  (family) | RepBase Name* | Subgroup and sequence source  (Genbank accession number) |
| --- | --- | --- | --- |
| Class I HERVs | HERV-I | HERVI | HERV-I (M92067) |
|  |  | HERVIP10F | HERV-IP-T47D (U27241) |
|  |  |  | Seq65 (AP000842) |
|  | HERV-T | HERVS71 | HERV-T(S71-TK6) (U12969) |
|  |  |  | HERV-T(S71-TK1) (U12970) |
|  | HERV-FRD | MER50I | HERV-FRD (U27240) |
|  |  |  | HS49C23 (Z93019) |
|  |  |  | HERV-Z (Z69907) |
|  | HERV-E | HERVE | HERV-E4-1 (M10976) |
|  |  |  | Seq32 (AC010636) |
|  | HERV-H | HERVH | RGH2 (D11078) |
|  |  |  | HERV-H (AF026252) |
|  |  |  | Seq66 (AL359740) |
|  | HERV-F | HERVH48I | HERV-F2 (AC002416) |
|  |  | HERVFH19I | HERV-F (Z94277) |
|  |  | HERVFH21 | HERV-Fb (AC000378) |
|  | HERV-W | HERV17 | HERV-W (AF009668) |
|  | HERV-R | HERV-R | ERV3 (AC004609) |
|  |  |  | HERV-Rb (AC004045) |
|  | ERV9 | HERV9 | Seq64 (AC005253) |
|  |  | HERV17 | Seq63 (AC018926) |
|  |  | HERV9 | ERV9 (X57147) |
|  |  | HERV9 | Seq59 (AC006397) |
|  |  | HERVFH19I | Seq60 (AL135749) |
| Class II HERVs | HERV-K(HML-1) | HERVK14I | HERV-K(HML-1) (U35102) |
|  |  |  | Seq29 (S77579) |
|  | HERV-K(HML-2) | HERVK | HERV-K10 (M14123) |
|  |  |  | HERV-K(HML-2.HOM) (U87592) |
|  |  |  | HERV-K(HP1) (U87588) |
|  |  |  | HERV-K(D1.2) (U87595) |
|  | HERV-K(HML-3) | HERVK9I | Seq26 (AC073115) |
|  |  |  | Seq34 (AL592449) |
|  |  |  | HERV-K(HML-3) (U35236) |
|  |  |  | HERV 1 (S66676) |
|  |  |  | Seq43 (AF047595) |
|  | HERV-K(HML-4) | HERVK13I | HERV-K-T47D (AF020092) |
|  | HERV-K(HML-5) | HERVK22I | HERV-K(HML-5) (U35161) |
|  | HERV-K(HML-6) | HERVK3I | HERV-K(HML-6) (U60269) |
|  |  |  | Seq38 (AC010328) |
|  |  |  | Seq56 (AC018558) |
|  | HERV-K(HML-7) | HERVK11DI | NMWV7 (AP003171) |
|  | HERV-K(HML-8) | HERVK11I | NMWV3 (AL513321) |
|  | HERV-K(HML-9) | - | NMWV9 (AC025569) |
|  | HERV-K(HML-10) | HERVKC4 | HERV-KC4 (U07856) |
|  |  |  | Seq31 (AL162734) |
| Class III HERVs | HERV-L | HERVL | HERV-L (G895836) |
|  |  |  | Seq39 (AC091914) |
|  |  |  | Seq45 (AC006971) |
|  |  |  | Seq51 (AL353741) |
|  |  |  | Seq58 (AL590730) |

* Genetic Information Research Institute, Sunnyvale, CA, USA, www.girinst.org [19]
