# Supplementary material for: HERV-E-Mediated Modulation of PLA2G4A Transcription in Urothelial Carcinoma
Source: PLoS One. 2012 Nov 7;7(11):e49341. doi: 10.1371/journal.pone.0049341 (PMC3492278; doi:10.1371/journal.pone.0049341)
Supplement: Table S2 — Comparison of HERV core transcription pattern in human tissues. (DOC) [file pone.0049341.s003.doc]

**Table S2.** Comparison of HERV core transcription pattern in human tissues

| HERV group | HERV subgroup | Uro-thelium  (n=6) | Brain  (n=12)  (Frank et al., [48]) | Mamma  (n=18)  (Frank et al., [56]) | Kidney  (n=14)  (Haupt et al., [57]) |
| --- | --- | --- | --- | --- | --- |
| **HERV-FRD** | Seq46 |  |  | x |  |
| **HERV-E** | HERV-E4-1 | x | x | x | **x** |
|  | Seq32 |  |  |  | **x** |
| **HERV-F** | HERV-F |  | x | x |  |
| **HERV-Rb** | HERV-Rb | x |  |  |  |
| **HERV-W** | HERV-W |  |  | x | **x** |
| **ERV9** | Seq63 |  |  |  | **x** |
|  | ERV9 | x | x | x | **x** |
|  | Seq59 |  | x | x | **x** |
| **HERV-K(HML-2)** | HERV-K10 |  |  | x | **x** |
|  | HERV-K  (HML-2.HOM) |  |  | x | **x** |
|  | HERV-K(HP1) |  | x | x | **x** |
|  | HERV-K(D1.2) |  | x | x | **x** |
| **HERV-K(HML-3)** | Seq26 |  |  |  | **x** |
|  | HERV-K(HML-3) |  |  | x | **x** |
| **HERV-K(HML-4)** | HERV-K-T47D | x | x | x |  |
|  | Seq10 |  | x | x |  |
| **HERV-K(HML-6)** | HERV-K(HML-6) |  | x | x | **x** |
|  | Seq38 |  | x | x |  |
|  | Seq56 |  |  | x |  |
| **HERV-K(HML-8)** | NMWV3 | x |  |  | **x** |
| **HERV-K(HML-9)** | NMWV9 |  | x |  |  |
| **HERV-K(HML-10)** | **HERV-KC4** | **x** | **x** |  |  |
